# Etiology and antibiotic resistance patterns of community-acquired urinary tract infections in J N M C Hospital Aligarh, India

**DOI:** 10.1186/1476-0711-6-4

**Published:** 2007-03-23

**Authors:** Mohammed Akram, Mohammed Shahid, Asad U Khan

**Affiliations:** 1 Interdisciplinary Biotechnology Unit, Aligarh Muslim University Aligarh 202002 India; 2 Microbiology Department, JN Medical College and Hospital, AMU, Aligarh India

## Abstract

**Background:**

Urinary tract infections (UTIs) remain the common infections diagnosed in outpatients as well as hospitalized patients. Current knowledge on antimicrobial susceptibility pattern is essential for appropriate therapy. Extended-Spectrum beta-Lactamase (ESBL) producing bacteria may not be detected by routine disk diffusion susceptibility test, leading to inappropriate use of antibiotics and treatment failure. The aim of this study was to determine the distribution and antibiotic susceptibility patterns of bacterial strains isolated from patients with community acquired urinary tract infections (UTIs) at Aligarh hospital in India as well as identification of ESBL producers in the population of different uropathogens.

**Methods:**

Urinary isolates from symptomatic UTI cases attending to the JN Medical College and hospital at Aligarh were identified by conventional methods. Antimicrobial susceptibility testing was performed by Kirby Bauer's disc diffusion method. Isolates resistant to third generation cephalosporin were tested for ESBL production by double disk synergy test method.

**Results:**

Of the 920 tested sample 100 samples showed growth of pathogens among which the most prevalent were *E. coli *(61%) followed by *Klebsiella *spp (22%). The majority (66.66%) of the isolates were from female while the remaining were from male. Among the gram-negative enteric bacilli high prevalence of resistance was observed against ampicillin and co-trimoxazole. Most of the isolates were resistant to 4 or more number of antibiotics. Forty two percent of isolates were detected to produce ESBL among which 34.42 % were *E. coli *isolates.

**Conclusion:**

This study revealed that *E. coli *was the predominant bacterial pathogen of community acquired UTIs in Aligarh, India. It also demonstrated an increasing resistance to Co-trimoxazole and production of extended spectrum β-lactamase among UTI pathogens in the community. This study is useful for clinician in order to improve the empiric treatment.

## Background

Urinary tract infection (UTI) is the second most common infectious presentation in community practice. Worldwide, about 150 million people are diagnosed with UTI each year, costing the global economy in excess of 6 billion US dollars [1]. UTI may involve only the lower urinary tract or may involve both the upper and lower tract. The term cystitis has been used to describe lower UTI, which is characterized by a syndrome involving dysuria, frequency, urgency and occasionally suprapubic tenderness. However, the presence of symptoms of lower tract without upper tract symptoms does not exclude upper tract infection, which is also often present [2].

UTIs are often treated with different broad-spectrum antibiotics when one with a narrow spectrum of activity may be appropriate because of concerns about infection with resistant organisms. Fluoroquinolone are preferred as initial agents for empiric therapy of UTI in area where resistance is likely to be of concern [3,4]. This is because they have high bacteriological and clinical cure rates, as well as low rates of resistance, among most common uropathogens [5-7]. The extensive uses of antimicrobial agents have invariably resulted in the development of antibiotic resistance, which, in recent years, has become a major problem worldwide [8].

The resistance pattern of community acquired UTI pathogens has not been studied extensively [5]. The etiology of UTI and the antibiotic resistance of uropathogenes have been changing over the past years, both in community and nosocomial infection [9,10]. However, there are not much information on etiology and resistance pattern of community acquired UTIs in India is available. This retrospective study was conducted to compare the frequency and drug resistance pattern in uropathogenes isolated from patients with community acquired UTIs in Aligarh, India as well as identification of ESBL producer strains among the uropathogens. This study is important for clinician in order to facilitate the empiric treatment of patients and management of patients with symptoms of UTIs. Moreover, the data would also help authorities to formulate antibiotic prescription policies.

## Materials and methods

### Sample collection and analysis

The study was conducted on patients attending outpatient clinics at the J.N.M.C.H, A.M.U., Aligarh between August 2004 and July 2005. Freshly voided midstream specimens of urine (n = 920) were submitted to the clinical microbiology laboratory of J.N.M.C.H, Aligarh for processing. Semi quantitative urine culture using a calibrated loop was used to inoculate blood agar and MacConkey plates [11]. Following the recommendations of Kass [12] in distinguishing genuine infection from contamination, significant monomicrobic bacteriuria was defined as culture of a single bacterial species from the urine sample at a concentration of >10^5 ^cfu/ml. Only a single positive culture per patient was included in the analysis. The significant pathogens were identified by standard biochemical procedures [13]. Hi-Media kits' manufacturer instructions were followed to identify species of these genera. Hi25™ Enterobacteriaceae identification kit and Hi E. coli™ Identification Kit were used. In addition Himedia Vogel-Johnson Agar was used for *S. aureus*, Hi media HiChrome ECC Agar was used for *E. coli *(Blue colony), *K. pneumoniae *(Rose pink colony) and *P. aeruginosa *(Star color colony). UTI patients included in this study were classified as young and middle aged (20–49 years), pediatrics (New born to 19 years) and elderly patients (50–80 years).

### Antibiotic susceptibility testing

Antimicrobial susceptibility testing was performed using the disk diffusion method as described by the National Committee for Clinical Laboratory Standards (presently called as Clinical Laboratory Standard Institute) [14]. Antimicrobial agents (disks) tested and reported were obtained from Hi-Media labs, Mumbai, India.

*E. coli *ATCC 25922, *S. aureus *ATCC 29213, *P. aeruginosa *ATCC 27853, *E. faecalis *ATCC 29212, *E. coli *BCC 2132 (ESBL producer), and *E. coli *ATCC 35218 (non-ESBL producer) were used as quality control strains. Interpretative criteria for each antimicrobial tested were those recommended by the NCCLS-2000 [14]

### ESBL Detection by NCCLS phenotypic method

The NCCLS-ESBL phenotypic confirmatory test with ceftazidime, cephotaxime, ceftriaxone and cefixime were performed for all the isolates by disk diffusion method on Mueller-Hinton agar plates with and without 10 μg of amoxyclav. Susceptibility test results were interpreted according to the criteria established by the NCCLS [15]. A ≥ 5-mm increase in the zone of diameter of third generation cephalosporins, tested in combination with amoxyclav versus its zone when tested alone was considered indicative of ESBL production. *E. coli *ATCC 25922 was used as ESBL negative and *K. pneumoniae *700603 was used are ESBL positive reference strain.

### Statistical analysis

To analyze the data it was reported in the form of diameter of inhibition zone during susceptibility testing of all bacterial isolates by disc diffusion test against different classes of antimicrobial agents. One-way ANOVA was performed to check the significant difference among the different groups. A difference was considered significant if the probability that chance would explain the results was reduced to less than 5% (p ≤ 0.05). The normality and homogeneity was also checked.

## Results

Of the 920 urine samples processed 100 (10.86%) gave significant growth of pathogens. The patients were between new born and 80 years of age. More cases of UTIs were recorded among young and middle age patients (20–49 years, 51.04%). Pediatric patients (new born to 19 years) comprised 36.45% and elderly (50–80 years) constituted 16.66 % of the total number. More organisms were isolated from women (66.66 %) than from men (33.34 %).

Age and gender wise data of prevalence of uropathogenes revealed that *E. coli *(87%) *Klebsiella pneumoniae *(89%) *Psueodomonas aeruginosa *(100%) and *Acinetobacter baumannii *(67%) infection was found to be more prevalent among middle aged female candidates as shown in table 1. Moreover, none of the *P. aeruginosa and A. baumannii *were found in pediatric patients (NB to 19 years) whereas *Acinetobacter *spps. were not observed among elderly patients (50–80 years) in our study (table 1).

**Table 1:** Age and gender wise distribution and frequency of uropathogenes isolated from community acquired infection

Age groups	Uropathogenes									
	
	*E. coli*		*K. pneu*		*S. aureus*		*P. aerog*		*A. baum*	
	
	M(%)	F(%)	M(%)	F(%)	M(%)	F(%)	M(%)	F(%)	M(%)	F(%)
NB-19 years	32	68	40	60	0	100	--	--	--	--
20–49 years	13	87	11	89	40	60	0	100	33	67
50–80 years	44	56	17	83	0	100	100	0	--	--

NB = New born, *K. pneu *= *Klebsiella pneumoniae*, *S. aureus = Staphylococcous aureus*, *P. aerog = Psueodomonas aeruginosa, A. baum = Acinetobacter baumannii*.

Of the 100 significant isolates, gram-negative aerobic rods accounted for 92 % while gram-positive cocci accounted for the remaining 8 % of the total pathogens. The frequency and distribution of the different microorganisms is summarized in Table 2. *E. coli *(61%), *K. pneumoniae*. (22%), *P. aeruginosa *(4.0%), *S. aureus *(7.0%), A. *baumannii*. (3.0%), *Citrobacter *spp. (2.0%) and *E. faecalis *(1.0%) were the most prevalent microorganisms in UTI patients. Frequency of UTIs was found more in elderly patients (51.04 %), principally women (66.66 %) than in pediatric patients.

**Table 2:** Frequency and resistance pattern of most frequently occurring UTI pathogens against 17 selected antimicrobial agents tested and % ESBL production.

Frequency and Distribution of Uropathogens		Percentage (%) of resistance to antimicrobial agent																	
Microorganisms identified	No. of isolates & percent (%) occurence	Cn	Ca	Ce	Ci	Cep	Cpm	G	Tb	Ak	Pc	I	Ao	Cf	Nx*	T	Co	Nf	ESBL producers (%)
													
		Cep^2^	Cep^3^				Cep^4^	Amn			P	C	M	Q	F		Others		

*E. coli*	61	69	65	56	55	85	67	64	73	51	84	0	75	69	69	76	76	80	34.42
*K. pneumoniae*	22	53	53	41	47	65	53	53	53	35	82	12	59	47	47	53	53	76	27.3
S. aureus	07	40	40	0	0	60	20	20	20	20	40	0	60	40	40	40	40	20	-
*P. aeruginosa*	04	67	67	67	67	100	67	67	67	33	67	0	67	33	33	100	100	100	-
*A. baumanni*i	03	67	67	33	33	67	33	33	33	0	67	0	67	0	0	33	33	33	-
p-values		0.551	0.054				0.021	0.0002			0.017	0.319		0.212	0.318	0.076	0.047		

Significant at p-value of < 0.05

* p value = 0.000

Ak = amikacin; Ao = aztreonem; C = chloramphenicol; Co = cotrimoxazole; Cpm = cefepime; Cep = cefpodoxime; Ca = ceftazidime; Ce = cephotaxime; Ci = ceftriaxone; Cn = cephoxitin; Cf = ciprofloxacin; G = gentamicin; Tb = tobramycin; I = imipenem; Pc = piperacillinl; T = tetracycline; Nf = nitrofurantoin; Nx = norfloxacin. Cep^2 ^= Second generation cephalosporins, Cep^3 ^= Third generation,Cep^4 ^= Fourth generation cephalosporins, Amn = Aminoglycosides, Mon = Monobactam, P = piperacillin, Q = Quinolones, F = Fluoroquinolones, C = Carbapenems, Others = T, Co and Nf.

The antimicrobial potency and spectrum for 17 selected antimicrobial agents of different classes against the five most frequent UTI pathogens are summarized in table 2. Among the β-lactum antibiotics, imipenem had the widest coverage against *E. coli *isolates (100%), followed by amikacin (49%), and extended spectrum cephalosporins (15–45%). Moreover, a high potency of the fluoroquinolones against *E. coli *was observed.

Our *Klebsiella *isolates showed high percent susceptibility against imipenem (88%) followed by amikacin and cephotaxime (59%) and ceftriaxone (53%). Nitrofurantoin, tetracycline, co-trimoxazole, and cefpodoxime were found to be highly resistant (100%) against *Pseudomonas *isolates. Among the beta lactam antibiotics, imipenem had the widest coverage against gram-negative isolates (100%). This was followed by the amikacin, ciprofloxacin and norfloxacin (67%).

Imipenem, amikacin, ciprofloxacin and norfloxacin showed highest percent susceptibility (100%) against *A. baumannii *in this study. Third and fourth generation cephalosporins were found to be more effective with significant percent susceptibility (67%) against *Acinetobacter *isolates. Aminoglycosides and macrolides also had the same percent susceptibility (67%). Whereas, all *Staphylococcus aureus *isolates were found to be susceptible against imipenem, ceftriaxone and cephotaxime. The high resistance rate (60%) against cefpodoxime, nalidixic acid and aztreonem was also observed among these isolates.

Age wise distribution of antibiotic resistance pattern revealed that uropathogens isolated from the patients of different age groups showed slight variation in percent resistance against different antibiotics. It was found that percent resistance against different generation of cephalosporin was found to be 60–80 % in pediatrics patients whereas 40–70% was observed among elderly and middle aged patients. Moreover, 2–3% isolates among middle aged and pediatric patients were resistant against imipenim while no resistance against imipenim was seen against elderly patients. Percent resistance against norfloxacin was quite high (74%) among pediatric patients compare to middle aged and elderly patients (55%). Rate of resistance against gentamycin was higher (75%) among pediatric patients than elderly and middle aged patients (50 %). Moreover, almost all of the isolates included in this study were found resistant to four or more antibiotics as shown in table 3. Twenty two different resistant patterns were observed among 61 *E. coli *strains. Each of these patterns was common in 1 or up to 3 strains. Moreover, an interesting finding was that pattern 2 was repeated in 26 *E. coli *strains as shown in table 3. Resistance patterns among *Klebsiella pneumoniae *was also varied, fourteen different patterns were observed among twenty two strains (Table 3).

**Table 3:** Resistance patterns of uropathogens isolated in this study

Uropathogens	Resistance Patterns	No. of isolates
*E. coli *:		
	1. Cep^2^, Cep^3^, Cep^4^, Amn, Mon, Pen, Qun, Fqn, T, Nf.	01
	2. Cep^2^, Cep^3^, Cep^4^, Amn, Mon, Pen, Qun, Fqn, T, Nf, Co.	26
	3. Cep^2^, Cep^3^, Cep^4^, Mon, Qun.	02
	4. Cep^4^, Mon, Pen, Qun.	01
	5. Pen, Amn.	02
	6. Cep^3^, Amn, Pen, Qun, Fqn, T, Nf, Co.	02
	7. Cep^3^, Amn, Pen, Qun, Fqn, Mon, Co.	02
	8. Cep^2^, Cep^3^, Cep^4^, Mon, Pen, T, Nf.	02
	9. Cep^3^, T, Nf, Co, Amn, Pen, Mon, Qun.	02
	10. Nf, Co, Amn, Mon, Pen, Qun, Fqn.	01
	11. T, Nf, Co, Amn, Pen.	02
	12. Cep^2^, Cep^3^, Cep^4^, Amn, Mon, Pen, Qun, Fqn, Nf.	02
	13. Cep^2^, Cep^4^, Qun, Nf.	01
	14. Cep^2^, Cep^3^, Cep^4^, Amn, Mon, Pen, Qun, T, Co.	03
	15. Pen, T, Nf, Co.	01
	16. Nf.	01
	17. Cep^3^, Cep^4^, Amn, Mon, Pen, Qun, Fqn, T, Nf, Co.	02
	18. Cep^2^, Cep^4^, Qun, Nf, T, Co.	01
	19. Cep^2, ^Amn, Pen, Qun, Fqn, T.	02
	20. Cep^3, ^Pen, Qun, Fqn, T, Co.	02
	21. Cep^2^, Cep^3^, Amn, Mon, Pen, Qun, Fqn, T, Co.	02
	22. Cep^4^, Mon, Qun, Nf.	01
*K. pneumoniae :*		
	1. Cep^2^, Cep^3^, Cep^4^, Amn, Mon, Pen, Qun, Fqn, T, Nf.	04
	2. Cep^3^, Cep^4^, Mon, Pen, Qun.	02
	3. Cep^2^, Amn, Mon, Pen, Qun, T, Nf.	02
	4. Pen, Fqn, T, Co.	01
	5. Pen, Nf.	01
	6. Cep^3^, Cep^4^, Amn, Mon, Pen, Qun, Fqn, T, Nf, Co.	02
	7. Cep^3^, Pen, T.	01
	8. Resistant to all the groups.	01
	9. Pens, Nf.	01
	10. Resistant to all except Car and T.	01
	11. Cep^2^, Cep^3^, Cep^4^, Car, Pen, T, Nf.	02
	12. Cep^2^, Cep^3^, Cep^4^, Mon, Pen, Nf.	02
	13. Resistant to all except Car.	01
	14. Cep^2^, Cep^3^, Cep^4^, Mon, Pen, Qun, Fqn, Nf, Co.	01
*P. aeruginosa*		
	1. Cep^2^, Cep^3^,Cep^4^, Amn, Mon, Pen, Qun, Fqn, T, Co, Nf.	01
	2. Cep^2^, Cep^3^, Cep^4^, Amn, Mon, Pen,Qun, T, Nf, Co.	02
	3. Cep^2^, Pen, Qun, T, Nf, Co.	01
*A. baumannii*		
	1. Cep^2^, Pen, T.	01
	2. Cep^2^, Cep^3^,Cep^4^, Mon, Amn, Pen, Qun, Co, Nf.	01
	3. Cep^3^, Qun, Mon.	01
*S. aureus*		
	1. Mon, Cep^3^, Amp, Qun, fqn,, Co.	02
	2. Cep^2^, Cep^3^, Pen, Qun, Fqn, T, Mon.	02
	3. Cep^3^, Mon, Qun.	01
	4. Cep^2^, Cep^4^,Pen, Fqn, Amn, T, Nf, Co.	02

Abbreviations:

Cep^2 ^= Second generation cephalosporins, Cep^3 ^= Third generation,Cep^4 ^= Fourth generation cephalosporins, Amn = Aminoglycosides, Mon = Monobactam, Pen = Penicillins, Qun = Quinolones,Fqn = Fluoroquinolones, Co = Cotrimoxazole, T = Tetracycline,Nf = Nitrofurantoin, Car = Carbapenems.

A total of 42% isolates were found to produce ESBL detected by the double disc diffusion test. Table 3 shows the frequency of ESBL producers for different UTI pathogens. Among the five most frequent UTI pathogens, *E. coli *(34.42 %) and *Klebsiella pneumoniae*. (27.3 %) were most prevalent ESBL producers. Other isolates were also ESBL producers but their numbers were insignificant as shown in table 2.

Statistical analysis revealed that the data obtained was obeying the normality as well as principle of homogeneity throughout, p-values were also calculated as indicated in Table 2.

## Discussion

This study shows the distribution and antibiotic susceptibility pattern of microbial species isolated from patients with community acquired UTIs in J.N.M.C.H, Aligarh. These organisms cause a variety of infections including UTIs [16]. In this study urinary samples of children were also included. A majority of pathogens were isolated from adult patients (51.04 %), principally women (66.66 %). It has been extensively reported that adult women have a higher prevalence of UTI than men, principally owing to anatomic and physical factors [8,17].

Antibiotic resistance is a major clinical problem in treating infections caused by these microorganisms. The resistance to the antimicrobials has increased over the years. Resistance rates vary from country to country [18]. Overall, isolates from Latin American countries show the lowest susceptibility rates to all antimicrobial agents followed by Asian-Pacific isolates and European strains. Strains from Canada exhibit the best global susceptibility testing results. (SENTRY Antimicrobial Surveillance Program, SASP) [18]. In our study, it accounted for approximately 61% of all clinically significant urinary isolates and 63% of all Enterobacteriaceae. This is consistent with the findings of previous studies in which *E. coli *was the predominant pathogen isolated from patients with community acquired UTIs [9,19]. However, *Klebsiella pneumoniae *are rarely encountered in cases of community-acquired UTI [8,9,20]. In the present study 22% of *Klebsiella *isolates were found to be present among all uropathogens studied. These isolates shows resistance against first generation cephalosporin, cephalothin, aminoglycosides, macrolides and lincosamides which is consistent with the previous data of other community- based studies [21].

Our *E. coli *and *Klebsiella *isolates are equally resistant to ampicillin (76% and 75% respectively) while for Co-trimoxazole, *E. coli *is more resistant (75%) than *Klebsiella *(53%) in this region. Indian isolates showed higher resistance against ampicillin and co-trimoxazole than the isolates from USA (39.1% and 18.6 % respectively) [22] and Europe (29.8% and 14.1% respectively) [23]. On the other hand, rate of resistance against these antibiotics in countries like Senegal (77% and 55%), Spain, (65% and 33%), Taiwan (80% and 56%), and Israel (66% and 26%) is comparable with Indian isolates [24-27].

In this study *E. coli *and *Klebsiella *isolatesare highly resistant against nitrofurantoin (80% and 76% resistant respectively). Whereas, this drug exhibited low resistance rate in the major part of the world (0–5.4%), despite of it's being used for many years [28]. This is probably due to the fact that this antibiotic has been widely used in treating community-acquired UTIs over the past decade in this region [29-31]. The resistance rate of *E. coli *to extended spectrum cephalosporins ranges from 55% to 85%, which is contrary to other community-acquired UTI studies in Europe, Israel and the US [27,29-32]. Higher resistance rate to all antibiotics used in this study with the exception of imipenem and amikacin may be explained as uncontrolled consumption of these antibiotics during the past decade in our region [29-31].

In the present study overall imipenem resistance was 12 % for *Klebsiella pneumoniae*, whereas, other isolates of uropathogens were found to be sensitive to imipenem. It is highly stable against β-lactamase and has an unusual property of causing a post antibiotic effect on gram-negative bacteria [33]. Due to its small molecular size it can over come the poor permeability of β-lactams for *Pseudomonas *by efficient penetration through the porin, OMP D [34]. Extended spectrum cephalosporins showed remarkable rates of resistance against *E. coli, K. pneumoniae, S. aureus, A. baumannii*, and *P. aeruginosa*. All *S. aureus *isolates were susceptible to third generation cephalosporins i.e. cephotaxime and ceftriaxone. Whereas, among *P. aerugonisa *all isolates showed resistance to cefpodoxime and cefuroxime. All *Acinetobacter *isolates were susceptible to amikacin and fluoroquinolones. The reported resistance varies from 10 to 30% in *P. aeruginosa*. and 3 to 10.3% in *A. baumannii*

In this study, 42 out of 100 isolates of UTI pathogens (42%) were found to produce ESBL. High prevalence rate of ESBL producing strains have also been reported earlier in *K. pneumoniae*. [35,6]. This is consistent with other drug resistance groups in India (48.3%) [7]. A 34.42 % of our *E. coli *isolates were ESBL producers, followed by 27.3% of *K. pneumoniae*. It might be possible that the high level of multi-drug resistance was most probably due to production of extended spectrum beta lactamases in these isolates [36-38]. More studies are required to know the exact magnitude of the problem in India.

## Conclusion

It is quite alarming to note that almost all of the isolates included in this study were found resistant to four or more antibiotics. Antibiotic resistance is becoming a big problem for the public health which threaten the lives of hospitalized individual as well as those with chronic conditions and add considerably to health care cost. Therefore, it is an important issue to be addressed by the policy makers to formulate a strict antibiotics prescription policy in our country. Moreover, this study concludes that *E. coli *and other isolates were more sensitive to imipenem and amikacin compared to the other antibiotics tested and therefore these may be the drugs of choice for the treatment of community-acquired UTIs in our region.

## Competing interests

The author(s) declare that they have no competing interests.

## Authors' contributions

MA has collected the sample and characterized bacterial isolates from UTI with the collaboration of MS. MA has performed all the experiments incorporated in this manuscript. AUK has designed the problem and guide throughout this study as well as helped in writing this manuscript. MA has written first draft of manuscript.

## References

[B1] Gonzalez CM, Schaeffer AJ (1999). Treatment of urinary tract infection: what's old, what's new, and what works. World J Urol.

[B2] Sobel JD, Kaye D, Mandell GL, Bennett JE, Dolin R (2000). Urinary tract infections. Mandell, Douglas and Bennett's Principals and practice of infectious diseases.

[B3] Schaeffer AJ (2002). The expanding role of fluoroquinolones. Am J Med.

[B4] Biswas D, Gupta P, Prasad R, Singh V, Arya M, Kumar A (2006). Choice of antibiotic for empirical therapy of acute cystitis in a setting of high antimicrobial resistance. Indian J Med Sci.

[B5] Goldstein FW (2000). Antibiotic susceptibility of bacterial strains isolated from patients with community-acquired urinary tract infections in France. Multicentre Study Group. Eur J Clin Microbiol Infect Dis.

[B6] Gupta V, Yadav A, Joshi RM (2002). Antibiotic resistance pattern in uropathogen. Indian J Med Microbiol.

[B7] Tankhiwale SS, Jalgaonkar SV, Ahamad S, Hassani U (2004). Evaluation of extended spectrum beta lactamase in urinary isolates. Indian J Med Res.

[B8] Kumar MS, Lakshmi V, Rajagopalan R (2006). Related Articles, Occurrence of extended spectrum beta-lactamases among Enterobacteriaceae spp. isolated at a tertiary care institute. Indian J Med Microbiol.

[B9] Manges AR, Natarajan P, Solberg OD, Dietrich PS, Riley LW (2006). The changing prevalence of drug-resistant Escherichia coli clonal groups in a community: evidence for community outbreaks of urinary tract infections. Epidemiol Infect.

[B10] Kahan NR, Chinitz DP, Waitman DA, Dushnitzky D, Kahan E, Shapiro M (2006). Empiric treatment of uncomplicated urinary tract infection with fluoroquinolones in older women in Israel: another lost treatment option?. Ann Pharmacother.

[B11] Beckford-Ball J (2006). Related Articles, Management of suspected bacterial urinary tract infection. Nurs Times.

[B12] Girou E, Rioux C, Brun-Buisson C, Lobel B (2006). Infection Committee of the French Association of Urology. The postoperative bacteriuria score: a new way to predict nosocomial infection after prostate surgery. Infect Control Hosp Epidemiol.

[B13] McNulty CA, Bowen J, Clark G, Charlett A, Cartwright K, South West G, Microbiology Laboratory Use Group (2004). How should general practitioners investigate suspected urinary tract infection? Variations in laboratory-confirmed bacteriuria in South West England. Commun Dis Public Health.

[B14] National Committee for Clinical Laboratory Standards (2000). Methods for Disk Susceptibility Tests for Bacteria That Grow Aerobically. NCCLS Documant M2-A7 Wayne, National Committee for Clinical Laboratory Standards.

[B15] National Committee for Clinical Laboratory Standards (2001). Performance standards for antimicrobial susceptibility testing. International Supplement NCCLS Committee for Clinical Laboratory Standards Wayne, Pa.

[B16] Meharwal SK, Taneja N, Sharma SK, Sharma M (2002). Complicated nosocomial UTI caused by nonfermenters. Indian J Urol.

[B17] Khan AU, Musharraf A (2004). Plasmid mediated multiple antibiotic resistance in *P. mirabilis *isolated from the UTI patients. Medical Sci Mon.

[B18] Gales AC, Jones RN, Turnidge J, Rennie T, Ramphal R (2001). Characterization of *Pseudomonas aeruginosa *isolates: occurrence rates, antimicrobial susceptibility patterns and molecular typing in the global SENTRY antimicrobial surveillance program 1997–1999. Clin Infect Dis.

[B19] Philippon A, Arlet G, Lagrange PH (1996). Escherichia coli: Frequency de resistance et evolution a divers antibiotiques urinaries dont la fosfomycine en milieu hospitalier. Med Mal Infect.

[B20] Kunin CM (1994). Urinary tract infections in females. Clin Infect Dis.

[B21] Dimitrov TS, Udo EE, Emara M, Awini F, Passadilla R (2003). Etiology and antibiotic susceptibility patterns of community-acquired urinary tract infections in Kuwait hospital. Med Princ Pract.

[B22] Vromen M, van der Van AJ, Knols AM, Stobberingh EE (1999). Antimicrobial resistance patterns in urinary tract isolates from nursing homes residents. Fifteen years of data reviewed. J Antimicrob Chemother.

[B23] Kahlmeter G (2003). Prevalence and antimicrobial susceptibility of pathogens in uncomplicated cystitis Europe. The ECO. SENS study. Int J Antimicrob Agents.

[B24] Dromigny JA, Nabeth P, Perrier Gros Claude JD (2002). Distrinution and susceptibility of bacterial urinary tract infections in Dakar, Senegal. Int J Antimicrob Agents.

[B25] Daza R, Gutierrez J, Piedrola G (2001). Antibiotic susceptibility of bacterial strains isolated from patients with community- acquired urinary tract infections. Int J Antimicrob Agents.

[B26] Lau SM, Peng MY, Chang FY (2004). Resistance rates to commonly used antimicrobilas among pathogens of both bacteremic and non-bacteremic community-acquired urinary tract infection. J Microbial Immunol Infect.

[B27] Colodner R, Keness Y, Chazan B, Raz R (2001). Antimicrobial susceptibility of community-acquired uropathogens in northern Israel. Int J Antimicrob Agents.

[B28] Honderlick P, Cahen P, Gravisse J, Vignon D (2006). Uncomplicated urinary tract infections, what about fosfomycin and nitrofurantoin in 2006?. Pathol Biol.

[B29] Mudur G (2000). Drug resistant cholera in India attributed to antibiotic misuse. BMJ.

[B30] Svetlansky I, Liskova A, Foltan V, Langsadl L, Krcmery V (2001). In creased consumption of fluoroquinolones is not associated with resistance in Escherichia coli and Staphylococcus aureous in the community. J Antimicrob Chemother.

[B31] Hillier SL, Magee JT, Howard AJ, Palmer SR (2002). How strong is the evidence that antibiotic use is risk factor for antibiotic-resistant, community -acquired urinary tract infection. J Antimicrob Chemother.

[B32] Farrel DJ, Morrisey I, De Rubeis D, Robbins M, Felmingham D (2003). A UK multi-center study of the antimicrobial susceptibility of bacterial pathogens causing urinary tract infection. J Infect.

[B33] Neu HC (1992). Resistance of *Pseudomonas aeruginosa *to imipenem. Infect Control Hosp Epidemiol.

[B34] El Amin N, Giske CG, Jalal S, Keijser B, Kronvall G, Wretlind B (2005). Carbapenem resistance mechanisms in Pseudomonas aeruginosa: alterations of porin OprD and efflux proteins do not fully explain resistance patterns observed in clinical isolates. APMIS.

[B35] Akata F, Tatman-Otcum M, Ozkan E, Tansel O, Otkum M, Tugrul M (2003). Prevalence of extended spectrum beta lactamases produced by nosocomial isolates of enterobacteriacae in Trakata University Hospital, Turkey. New Microbial.

[B36] Grover SS, Sharma M, Chattopadhya D, Kapoor H, Pasha ST, Singh G (2006). Phenotypic and genotypic detection of ESBL mediated cephalosporin resistance in Klebsiella pneumoniae: emergence of high resistance against cefepime, the fourth generation cephalosporin. J Infect.

[B37] Mathai D, Rhomberg PR, Biedenbach DJ, Jones RN, India Antimicrobial Resistance Study Group (2002). Evaluation of the in vitro activity of six broad-spectrum beta-lactam antimicrobial agents tested against recent clinical isolates from India: a survey of ten medical center laboratories. Diagn Microbiol Infect Dis.

[B38] Mohanty S, Singhal R, Sood S, Dhawan B, Das BK, Kapil A (2005). Comparative in vitro activity of beta-lactam/beta-lactamase inhibitor combinations against gram negative bacteria. Indian J Med Res.

